# Under-Appreciated Phylogroup Diversity of *Escherichia coli* within and between Animals at the Urban-Wildland Interface

**DOI:** 10.1128/aem.00142-23

**Published:** 2023-05-16

**Authors:** Katherine M. Lagerstrom, Elizabeth A. Hadly

**Affiliations:** a Department of Biology, Stanford University, Stanford, California, USA; b Jasper Ridge Biological Preserve, Stanford University, Stanford, California, USA; c Center for Innovation in Global Health, Stanford University, Stanford, California, USA; INRS Armand-Frappier Sante Biotechnologie Research Centre

**Keywords:** *Escherichia coli*, phylogroup diversity, wild animals, rarefaction, nested analysis, anthropogenic impacts

## Abstract

Wild animals have been implicated as reservoirs and even “melting pots” of pathogenic and antimicrobial-resistant bacteria of concern to human health. Though Escherichia coli is common among vertebrate guts and plays a role in the propagation of such genetic information, few studies have explored its diversity beyond humans nor the ecological factors that influence its diversity and distribution in wild animals. We characterized an average of 20 E. coli isolates per scat sample (*n* = 84) from a community of 14 wild and 3 domestic species. The phylogeny of E. coli comprises 8 phylogroups that are differentially associated with pathogenicity and antibiotic resistance, and we uncovered all of them in one small biological preserve surrounded by intense human activity. Challenging previous assumptions that a single isolate is representative of within-host phylogroup diversity, 57% of individual animals sampled carried multiple phylogroups simultaneously. Host species’ phylogroup richness saturated at different levels across species and encapsulated vast within-sample and within-species variation, indicating that distribution patterns are influenced both by isolation source and laboratory sampling depth. Using ecological methods that ensure statistical relevance, we identify trends in phylogroup prevalence associated with host and environmental factors. The vast genetic diversity and broad distribution of E. coli in wildlife populations has implications for biodiversity conservation, agriculture, and public health, as well as for gauging unknown risks at the urban-wildland interface. We propose critical directions for future studies of the “wild side” of E. coli that will expand our understanding of its ecology and evolution beyond the human environment.

**IMPORTANCE** To our knowledge, neither the phylogroup diversity of E. coli within individual wild animals nor that within an interacting multispecies community have previously been assessed. In doing so, we uncovered the globally known phylogroup diversity from an animal community on a preserve imbedded in a human-dominated landscape. We revealed that the phylogroup composition in domestic animals differed greatly from that in their wild counterparts, implying potential human impacts on the domestic animal gut. Significantly, many wild individuals hosted multiple phylogroups simultaneously, indicating the potential for strain-mixing and zoonotic spillback, especially as human encroachment into wildlands increases in the Anthropocene. We reason that due to extensive anthropogenic environmental contamination, wildlife is increasingly exposed to our waste, including E. coli and antibiotics. The gaps in the ecological and evolutionary understanding of E. coli thus necessitate a significant uptick in research to better understand human impacts on wildlife and the risk for zoonotic pathogen emergence.

## INTRODUCTION

Escherichia coli is a common commensal of the vertebrate gut and is highly genetically diverse and widely geographically distributed. The phylogeny of E. coli consists of 8 major phylogroups: A, B1, B2, C, D, E, F, G, and cryptic Escherichia clades I to V (1–3). These phylogroups are known to differ in their phenotypic and genotypic characteristics, lifestyles, ecological niches, ability to cause disease, and propensity to harbor antimicrobial resistance (AMR) genes (4), yet the underlying mechanisms of these differences remain largely unknown. Preeminently, this is due to the enormous genetic diversity encompassed by E. coli (5). The average E. coli genome consists of around 4,700 genes, whereas the pangenome, or total gene pool across the species, continues to rise as more genomes are sequenced, but has been estimated to contain anywhere from 43,000 gene families (6) to essentially an infinite number (7); somewhere between 1,000 and 3,000 genes of these are thought to be common to all E. coli (8). Despite pervasive horizontal gene transfer (HGT) and homologous recombination (HR) that make gene conversion 100 times more likely than mutation at an individual nucleotide (5), the phylogroups remain robust (9), with intra-group recombination being more prevalent than inter-group recombination (10). Variations in genome size are associated with phylogroup and isolation source, and mobile genetic elements play a strong role in both, suggesting that gene flow reinforces the associations between phylogroup and habitat (11).

There is a growing body of evidence that the phylogroups also differ in their transmission dynamics and competitivity (12). For example, when phylogroup B2 is present, the host tends to have lower phylogroup diversity overall than hosts harboring other phylogroups; this has been attributed to virulence-associated traits that may enhance its fitness in the host gut (13). Phylogroups A, B2, and D are typically most common in humans (4, 14), with significant geographical variation. Phylogroup B1 is prevalent in animals and the environment and carries genetic factors that facilitate adaptation to soil, water, and even plant colonization (15). Phylogroups B2, D, and F encompass the majority of extra-intestinal pathogenic strains, while phylogroups A, B1, and C contain most of the intestinal pathogenic E. coli responsible for dysentery and hemolytic uremic syndrome in humans (16–18). The more recent classifications, phylogroups F and G, contain highly virulent and AMR strains (2, 19). Of the cryptic clades, clade I is associated with disease in humans, while clades II to V are thought to potentially be of environmental origin and therefore more adapted to life outside a host (20), although there is evidence that they also circulate in birds and nonhuman mammals (21).

The presence of AMR in wild animals is a growing concern (22), as is the role of wild animals as reservoirs of pathogenic E. coli (23). Thus, most previous studies on E. coli in wildlife have targeted pathogenic and AMR strains, thereby overlooking substantial genetic diversity because most E. coli are commensal (24). We cannot fully comprehend the risks of wildlife harboring AMR and pathogenic strains of E. coli without understanding the underlying eco-evolutionary dynamics of commensal E. coli, especially since pathogenic strains of E. coli can evolve from harmless lineages (25). One of the first studies to examine commensal E. coli in wild animals, in 1999, found the then-highest genetic diversity of any sample studied (26). They concluded that geographic effects and host taxonomy accounted for most of this genetic differentiation; however, these results were obtained by sampling a single isolate per host, thus likely still overlooking substantial diversity. A broad survey of over 2,300 vertebrate hosts in Australia showed many significant results, including that animals living in proximity to humans were more likely to harbor E. coli, and that the relative abundances of the 4 major phylogroups (A, B1, B2, and D) depended on climate, host diet, and body mass, but here too, only a single colony was selected per fecal sample (27). Notably, in a recent comprehensive investigation of AMR E. coli in the synanthropic Australian silver gull (Chroicocephalus novaehollandiae), isolates carrying clinically relevant resistance were not confined to specific phylogroups, but rather spanned all major phylogroups (28). This substantiates the need to cast a broader net by sampling multiple colonies per individual host when seeking to understand the genetic diversity and eco-evolutionary dynamics of E. coli in wild animals.

**FIG 1:**
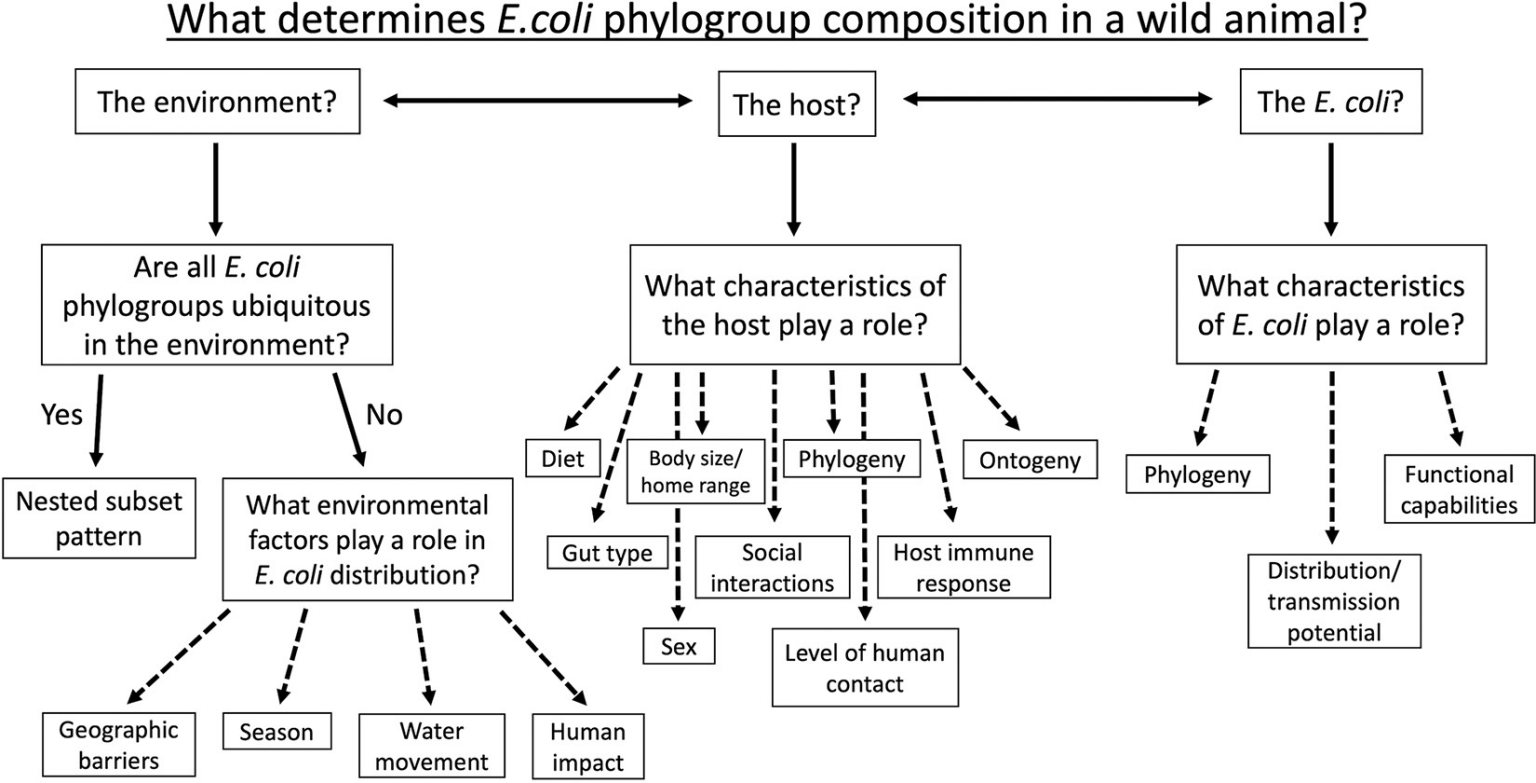
Potential determinates of Escherichia coli phylogroup composition. A flow diagram outlining potential factors influential to determining the community structure of the phylogroups within a host related to the environment, the host, and E. coli itself.

How E. coli colonizes a host is likely influenced by many different factors associated with the environment, the host, and the E. coli itself (Fig. 1). Indeed, a recent review assessed such contributing factors in humans as environmental exposure, temporal dynamics, and various forms of intraspecific competition between E. coli strains (29). Some factors contribute to the apparent host-generality of E. coli, including shared environments (e.g., water sources), predator-prey relationships, and close association between hosts (e.g., humans and domestic animals [30]). Several studies also support a role for host adaptation and thus some level of host-specificity in E. coli (30–33). However, they often conclude that such studies, as well as the ability to source-trace and predict pathogenic E. coli contamination, would be greatly enhanced by obtaining genomes from a wider range of animal hosts and environments (33). Because E. coli persists in human, animal, and environmental reservoirs, and can cause morbidity in a range of host species, it extremely important for us to expand and deepen sampling efforts across this range to understand the distribution, diversity, and evolutionary trajectory of E. coli (24). This need is made more urgent by the rapidly changing environments and level of human impact defining the Anthropocene.

Previously, most studies on E. coli in wildlife have relied on a single representative isolate per individual host under the assumption that this represents the within-host phylogroup diversity due to the relatively stable clonal structure of commensal E. coli (34). However, it is known that humans host an average of 3.5 resident phylogroups and many more transiently (14, 35). Gaining a better understanding of which factors contribute to the community assembly of E. coli within a host, including potential routes of transmission and acquisition, will require comprehensive sampling of individual hosts. Here, we tested 161 scat samples for E. coli from 14 wild and 3 domestic species at a biological preserve in California and characterized an average of 20 E. coli isolates per positive sample. In doing so, we captured all globally known phylogroup diversity from a single animal community in a small geographic area. We show that the relative abundances of the phylogroups vary within and across host species and assess the contributions of various host and environmental factors to determining within-host phylogroup composition.

## RESULTS

### Sampling host species to phylogroup-level saturation.

**FIG 2:**
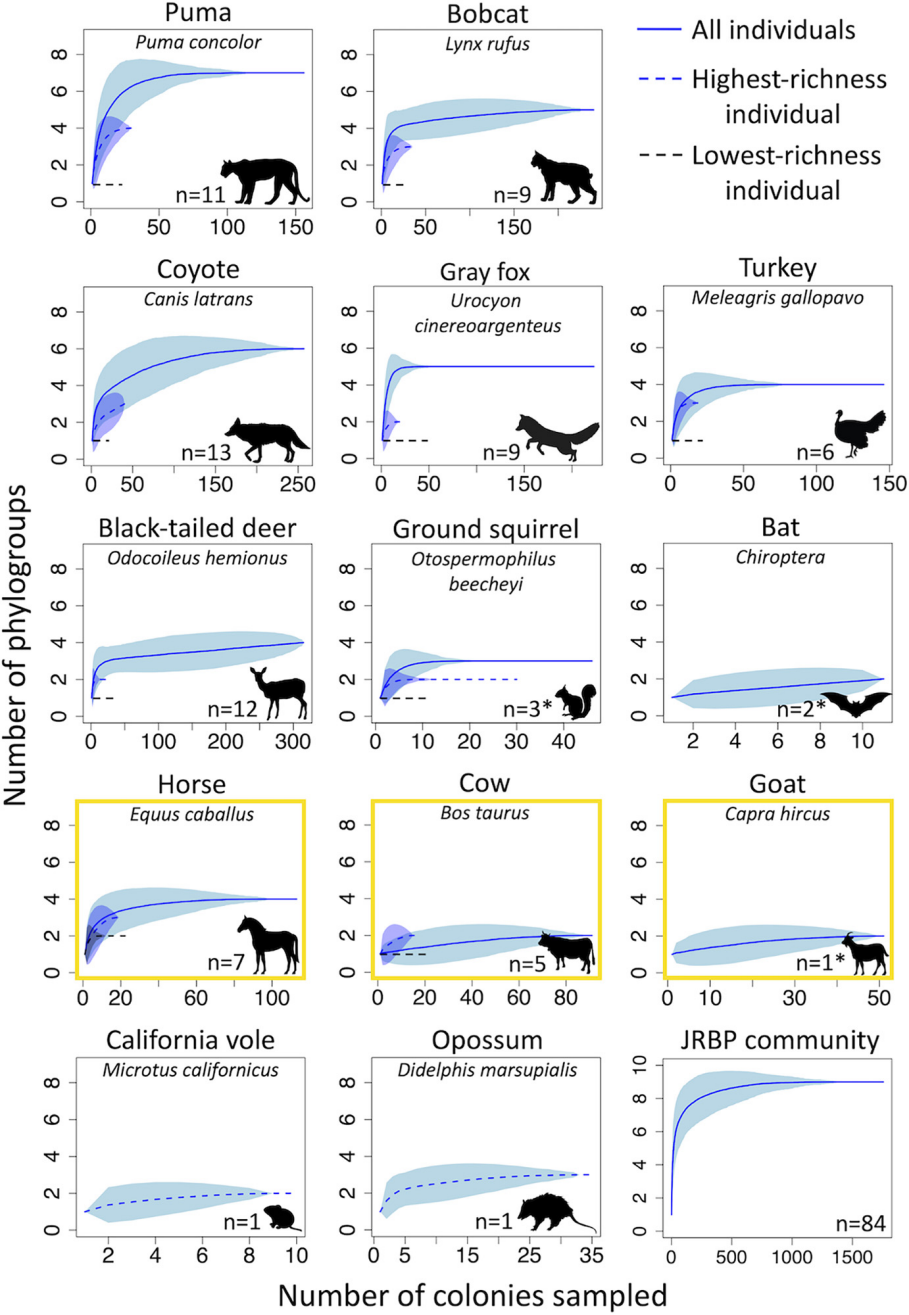
Sampling host-associated E. coli to phylogroup-level saturation. Rarefaction curves for the number of E. coli colonies necessarily sampled to reach phylogroup-level saturation for each host species. Not shown are species with only 1 phylogroup present (*n* = 1, including Lagomorpha, dusky-footed woodrat, long-tailed weasel, and western fence lizard). Dotted blue lines: saturation curve for the richest scat sample of a host species. Dotted black lines: saturation curve for the least rich individual. Yellow outlines indicate the domestic host species. Asterisks denote multi-individual samples.

Escherichia coli was present in 52% of the 161 scats sampled (Table S1). Similar results were obtained in a previous investigation of the prevalence of E. coli in wild animals, where E. coli was detected in 56% of 1,063 mammalian hosts (27). Additionally, in agreement with this study, the average body mass of the host species sampled here positively correlated with E. coli prevalence by simple linear regression (Fig. S1 in the supplemental material; R^2^ = 0.68, *P < *0.001). However, in contrast to the previous study, which found the lowest prevalence of E. coli in carnivores, we found the highest prevalence in carnivores (60%) and the lowest prevalence in herbivores (46%), with omnivores falling in between (54%; Table S1). Total phylogroup richness encompassed by a host species differed among host species, as did the phylogroup richness encapsulated by different individuals (Fig. 2). All host species sampled to *n* > 2 individuals reached saturation above 1 phylogroup, except for cows, which were not sampled to saturation. The saturation levels for each host species were as follows: puma, 7 phylogroups; coyote, 6; bobcat and gray fox, 5; turkey, black-tailed deer, and horse, 4; and ground squirrel, 3. Small mammals were not sampled to saturation due to the opportunistic sampling methods used here and therefore will require more extensive sampling efforts. However, the individual California vole and opossum samples each carried more than 1 phylogroup (2 and 3, respectively). Conversely, a single phylogroup was isolated multiple times from each of the woodrat, rabbit, lizard, and weasel scats. Fifty-one isolates were sampled from the goat community, and all belonged to phylogroup B1 except for 2 from phylogroup A, suggesting that the community may not have been sampled to saturation. The horse was the only host species for which every individual (*n* = 7) carried more than 1 phylogroup. The average number of phylogroups per individual scat across all samples was 1.8 and the average number isolated from each host species was as follows: puma, 2.18; bobcat, 2; coyote, 1.85; turkey, 1.67; black-tailed deer, 1.58; gray fox, 1.44; ground squirrel, 1.3; horse, 2.7; goat, 2; and cow, 1.2 (Fig. S2a). Rarefactions on the individual scat samples with more than 1 phylogroup present estimated that 73% were sampled to saturation. The diversity of E. coli in the single opossum sample and an individual turkey sample were exceptionally high when considering that each phylogroup has multiple banding patterns associated with it (i.e., different combinations of marker genes), as the opossum carried 5 and turkey carried 6 distinct patterns (Fig. S2b). Within-sample phylogroup richness did not differ between host diet types (Fig. S3b). Of the wild species sampled to saturation (puma, bobcat, coyote, gray fox, deer, turkey, and ground squirrel), the average individual home range size of each host species positively correlated with the phylogroup saturation level by simple linear regression (Fig. S4; R^2^ = 0.78, *P < *0.01), suggesting that hosts with larger ranges sample a greater diversity of E. coli, perhaps via exposure to a greater diversity of environments. We could not assess the relationship between status (wild or domestic) and phylogroup richness because horses were the only domestic species sampled to saturation and because the home ranges of domestic animals are governed by humans.

### Phylogroup diversity within and between individual hosts.

**FIG 3:**
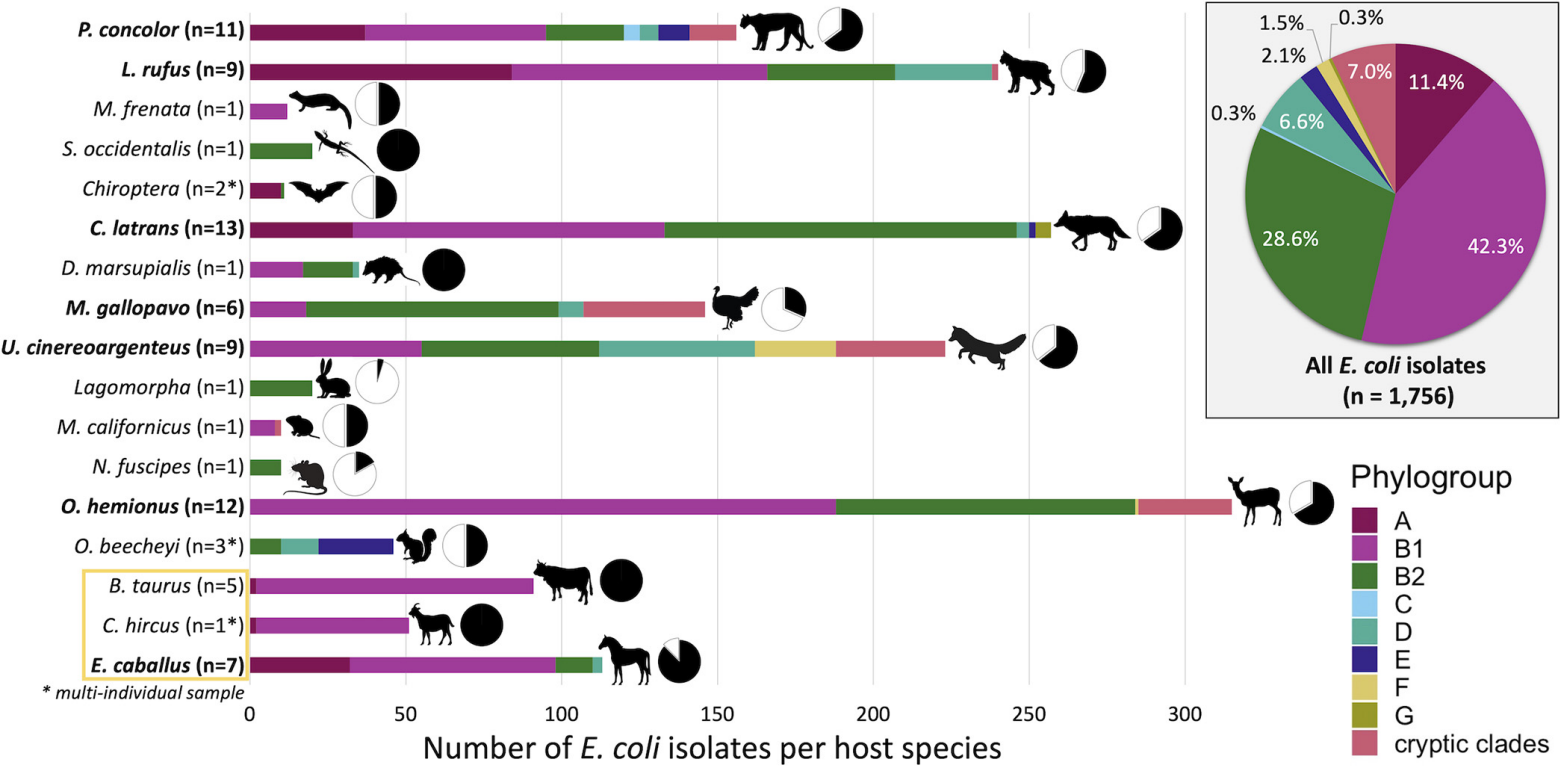
Isolation source and phylogroup diversity of E. coli from Jasper Ridge Biological Preserve. For those scat samples yielding E. coli, bars show the number of E. coli isolates obtained from each host species, colored by phylogroup. Bold text indicates host species that were sampled to phylogroup-level saturation and the *n* value next to the host species represents the number of scat samples that contributed E. coli isolates. Small pie charts next to the host species’ silhouettes indicate the proportion of total scat samples that yielded E. coli. Domestic host species are indicated in yellow. Large pie chart illustrates the proportion of each of the 8 phylogroups and cryptic clades encompassed by 1,756 isolates from 84 scat samples.

**FIG 4:**
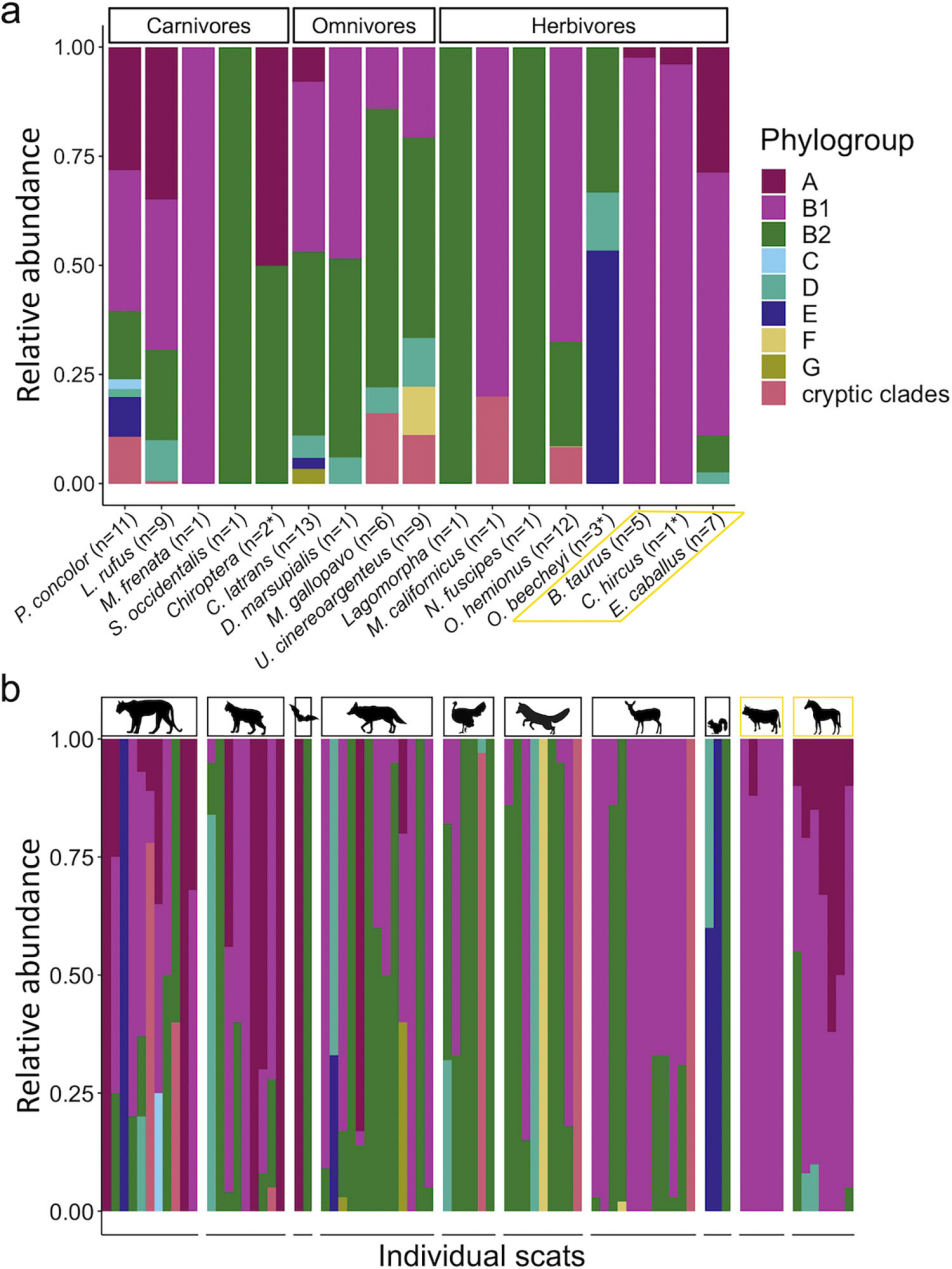
E. coli phylogroup diversity within and between hosts. (a) Species-level phylogroup diversity shown as the proportion of the total number of isolates sampled per host species. (b) Individual-level phylogroup diversity, grouped by host species; species with *n* = 1 are not shown. Yellow lines distinguish the domestic host species.

Escherichia coli was not consistently present among individuals within each host species (Fig. 3, see pie charts next to the hosts’ silhouettes) and the phylogroups were differentially abundant across host species (Fig. 3). All 8 phylogroups were recovered from the animal community at the Jasper Ridge Biological Preserve (JRBP), with B1 being the most prevalent (42.3%), followed by B2 (28.6%) and A (11.4%). Rarer groups included the cryptic clades (7.0%) and phylogroups D (6.6%), E (2.1%), F (1.5%), G (0.3%), and C (0.3%; Fig. 3). Phylogroup diversity varied greatly among host species (Fig. 4a) and among individuals of the same species (Fig. 4b) measured by the relative abundance of each phylogroup within all samples of a host species combined, and within each individual sample, respectively. Interestingly, the domestic animals, although herbivores, had a different pattern of phylogroup abundance from that of their wild counterparts. Phylogroup A was never isolated from a wild herbivore; however, it was present in ~70% of domestic animal samples (all of which were herbivores). Additionally, the relative abundance of phylogroup B2 was significantly higher in wild herbivores than in domestic herbivores (Fig. S6c; Wilcoxon, *P < *0.05). We also observed a significant difference in the relative abundance of phylogroup A in wild hosts across diet types (Fig. S5a; Kruskal-Wallis, *P < *0.001), as wild carnivores carried phylogroup A more often than omnivores or herbivores. Phylogroup A was also significantly more abundant in *Felidae* (Wilcoxon, *P < *0.05) and domestic horses (Wilcoxon, *P < *0.001) compared to *Canidae* (Fig. S7a). The significance of other such comparisons could not be calculated due to the low sample sizes for some host species and because there was substantial dispersion in phylogroup abundance across samples within a host species (Fig. S7b and c). No significant differences were observed in the relative abundances of the other dominant phylogroups (B1 and B2) across diet types of wild hosts (Fig. S5b and c), nor was the relative abundance of phylogroup B1 significantly different between wild and domestic herbivores (Fig. S6b).

Although phylogroup D is often considered a predominate phylogroup along with A, B1, and B2, it was found at relatively low prevalence here (6.6% of all isolates), and only in 1 puma, 1 bobcat, 2 horses, 1 ground squirrel, the opossum, 1 coyote, and 2 turkeys. Phylogroup E was only found in 1 puma, 1 coyote, and 2 ground squirrel samples. Phylogroup F composed 100% of isolates (*n* = 26) from 1 gray fox, and just 1 of 54 (1.8%) from a deer. Phylogroup C was the rarest, only isolated from 1 puma sample at 25% abundance (4 of 20 isolates). Phylogroup G was also very rare, only identified at low prevalence in 2 coyote samples, 1 of 40 (2.5%) and 4 of 10 (40%) isolates, respectively.

### Distribution of phylogroups in the environment and host sampling ability.

Because the rarest phylogroups were only isolated at very low abundance after sampling enough colonies to saturate the phylogroup discovery curve for a host species, we further hypothesized that all phylogroups could persist in any host and the probability of their detection was a function of their abundance in the environment, the depth at which a host samples its environment, and the depth at which that host is sampled in the lab. Nested subset analysis (“nestedness”) is a way to investigate whether the same species pool (i.e., E. coli phylogroups) is available for all hosts to sample in the environment (36). In a nested community, common E. coli phylogroups would be found in most hosts while rare phylogroups would only be found in the hosts with high phylogroup diversity. A nested subset analysis on the aggregated presence/absence matrix of phylogroups grouped by host species resulted in a significant nested signal (Fig. S8a), but Spearman’s test revealed that the nestedness rank (Fig. S8b) was highly correlated with the number of individuals sampled per species (Spearman’s ρ = −0.9; *P < *0.0001). To correct for variation in the number of individuals sampled per species, we generated 1,000 matrices of 1 randomly selected representative individuals per each host species and ran the analysis again. This resulted in the absence of evidence for a nested community, although interestingly, the single opossum was consistently ranked lowest and cows the highest (Fig. S8b). Separating the nested signal into E. coli versus host species, the E. coli phylogroup was less nested than random (*P < *0.05) 12% of the time but never more nested than random, whereas host species was never less nested than random but was more nested 2.9% of the time. The full matrix was never less nested than random and was more nested just 0.7% of the time.

### Influence of host and environmental variables on within-sample phylogroup composition.

**FIG 5:**
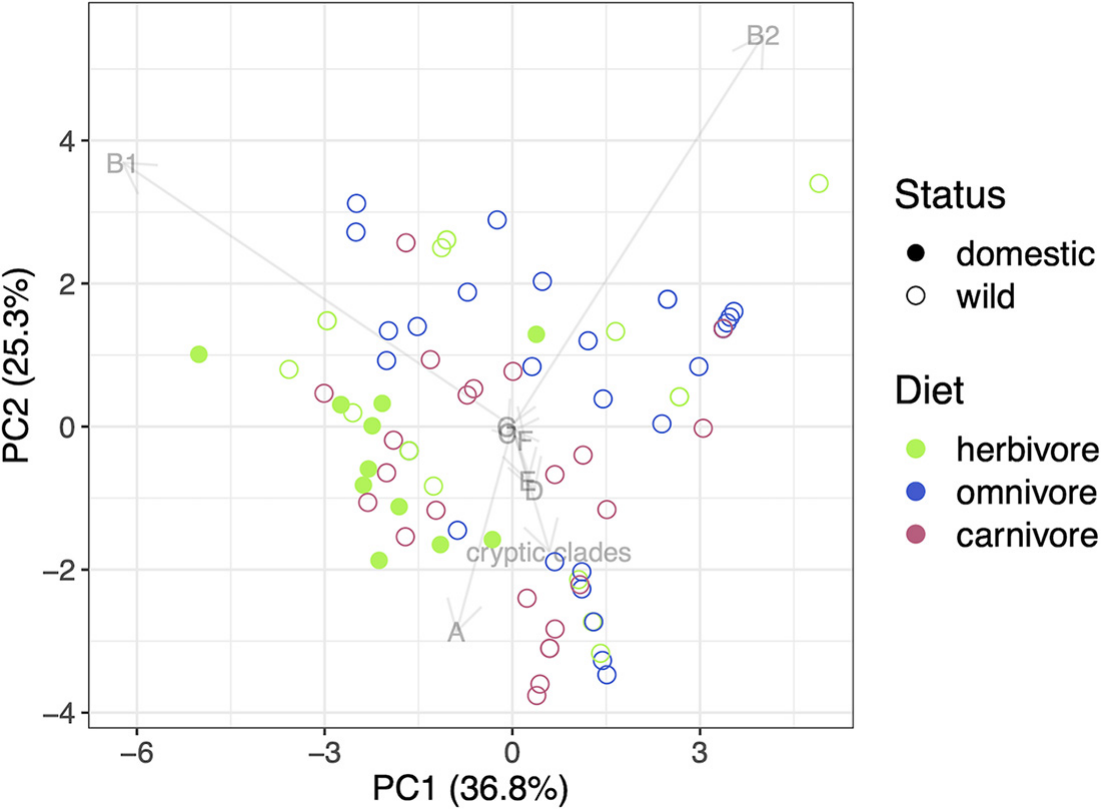
Two-dimensional principal-component analysis (PCA) plot of within-sample E. coli phylogroup composition. Each point corresponds to the phylogroup composition of an individual scat sample in the first two principal components (PC). Percentages in the axis labels correspond to the amount of variation explained by the PC. Samples from domestic hosts are shaded to distinguish these from samples from wild hosts; colors correspond to the host species’ diet type. Eigenvectors for the phylogroups in the first two PC axes are shown.

The first two principal components (PC) of the principal-component analysis (PCA) together accounted for 62% of the variance across within-sample phylogroup composition, but samples did not visually appear to cluster based on host diet type or other factors as well as they did by host status (Fig. 5). The resulting eigenvectors for each of the phylogroups in PC 1 and 2 suggest that phylogroups A, B1, and B2 are less likely to co-occur with one another, an observation supported by previous findings that phylogroup B2 is competitively dominant in humans (35, 37) and that the occurrence of phylogroups A and B1 is negatively correlated (38). Permutational multivariate analyses of variance (PERMANOVA) of the differences between within-sample E. coli phylogroup composition and host and environmental factors identified host status (wild or domestic) and season (wet or dry) at the time of collection as significant variables, with host status having the largest effect, explaining 43.24% of the total variation (Table 1; PERMANOVA, *P = *0.006). When accounting for the nesting of host species within diet type, and diet type within host status, these host variables were not significant determinants of within-sample phylogroup composition. Additionally, within-sample phylogroup composition did not significantly vary across the 5 years of sample collection, nor did the age assigned to a scat sample upon collection influence within-sample phylogroup composition.

**TABLE 1:** PERMANOVA results evaluating the influence of host and environmental factors on E. coli phylogroup composition within individual scat samples^*a*^

Sample characteristic	*df*	SS	MS	Pseudo-F	*P* (perm)	Estimated variation (%)
Scat age	2	28.28	14.14	1.33	0.221	
Host status	1	84.49	84.49	9.24	**0.006**	43.24
Season (wet/dry)	1	28.75	28.75	2.71	**0.027**	7.19
Collection yr	4	60.81	15.20	1.43	0.111	
Diet (status)	2	16.93	8.47	0.77	0.616	
Host species (diet [status])	13	147.84	11.37	1.07	0.333	
Residuals	60	637.26	10.62			49.57
Total	83	1,056.5				

a PERMANOVA, permutational multivariate analysis of variance; *df*, degrees of freedom; SS, sum of squares; MS, mean squares. Variation estimates are reported for statistically significant variables (in bold).

## DISCUSSION

The within-host diversity of E. coli has been well-studied in humans (39); however, wild animals have been starkly overlooked in these investigations despite their likely ability to carry a significant amount of diversity, which elicits fear of the generation of more pathogenic and AMR strains *in situ* via HGT and HR. We identified all defined E. coli phylogroups in animals residing at a small preserve in California and found that 57% of samples contained more than 1 phylogroup, often at relatively equal abundances. By sampling multiple isolates per individual and multiple individuals per species, we acquired a greater number of phylogroups, supporting an effect of sampling intensity on the total phylogroup richness obtained. We illustrated this effect with nested subset analyses, which was significant on aggregated host species phylogroup-level diversity, but insignificant after controlling for variation in our sampling depth across host species. We show that the relative phylogroup abundances differed across host species and individuals and suggest that within-host phylogroup composition is likely influenced by a combination of multiple interacting host and environmental variables. These could include associations between the animal food web, social interactions, microbiome assembly processes, environmental habitats, and human impacts (11).

Why is the global E. coli phylogroup diversity present in such a small geographic area? One possible explanation is that E. coli phylogroup distribution follows a nested pattern that is also influenced by a variety of host and E. coli characteristics, as well as environmental factors. For example, different life histories of E. coli may contribute to the differential abundance of phylogroups or variation in the bacterial species pool in certain environments, which may explain why certain phylogroups, like C and G, are extremely rare. Previous studies have indicated, and we confirmed here, that there is a positive relationship between host body mass and E. coli prevalence across host species (i.e., the bigger you are, the more likely you are to carry E. coli [24]). We expand on this to suggest that host species with a larger individual home range, noting that body mass and individual home range size co-correlate in mammals (40), should also carry greater E. coli phylogroup diversity. In support of this hypothesis, we showed that the average individual home range of a host species positively correlated with host species-level phylogroup richness. If E. coli phylogroup diversity is globally distributed and nested, then we should be able to sample a large enough number of individuals from any given host species to obtain all known E. coli phylogroups, and therefore any host specificity or preference must be occurring at a genetic level below the phylogroup. Apart from the need to sample even more individuals per host species, another explanation for the lack of a strong nested signal here is that phylogroup composition might be more strongly influenced by host and E. coli characteristics than by the distribution of E. coli in the environment, such that hosts may have the ability to differentially reject or accept certain E. coli phylogroups, or that exclusionary processes or competitive interactions are occurring between the phylogroups themselves. These hypotheses have yet to be well-investigated; however, a recent study found a strong negative correlation between phylogroups A and B1 in septic tanks which may indicate intraspecific competition (38). It is likely that many factors are involved in determining the E. coli phylogroup composition within a host, although, as in most ecological studies of diversity and abundance, the ability to accurately characterize E. coli diversity is directly related to the number of colonies sampled and the underlying prevalence of the strain (41).

Many questions remain surrounding the significance of individual wild animals carrying diverse E. coli phylogroups, but it could indicate the level of human impact on wildlife populations via waste pollution because E. coli is transmitted primarily via the fecal-oral route and has commonly been used as an indicator of fecal contamination in water and agriculture (42). The detection of phylogroup G isolates in coyotes here is significant due to its high propensity to be virulent and resistant to multiple antibiotics (2), indicating coyotes as potential reservoirs for pathogenic and AMR E. coli. This is potentially attributable to their lifestyle (omnivorous and coprophagic) and closer proximity to human populations. Gray foxes were nearly the sole reservoir of phylogroup F, which contains several sequence types capable of causing disease in humans, pets, livestock, and wild birds, and is also likely to carry resistance to fluroquinolone antibiotics (19). Phylogroup C was the rarest in our study, only isolated from 1 puma sample. Pumas also have the largest individual home ranges of any species in this study, perhaps indicating that an individual puma picked up an E. coli strain belonging to phylogroup C at a site outside the preserve. The same could be true for the rare occurrence of phylogroup G in coyotes, which are known to frequent more residential areas outside the preserve. The increased abundance of phylogroup A in domestic species could be due to their direct exposure to antibiotics, as phylogroup A has been suggested to have a genetic background primed for AMR gene development (43). Among the wild animals, phylogroup A occurred at the highest prevalence in carnivores, which could indicate that they face stronger anthropogenic pressure through exposure to a wider range of environments and dietary diversity, or that AMR E. coli accumulate across trophic levels. It could also be due to other functional differences between phylogroups, for which more comprehensive genetic studies are needed. Although it has previously been suggested that domestic animals carry lower E. coli diversity than wild animals (44), this was not supported at the level of phylogroup diversity assessed in this study. Additionally, previous studies have provided evidence that the hindgut complexity favors B2 strains (27); however, phylogroup B1 was the predominant strain in horses, followed by phylogroup A. Previous studies have also suggested a higher prevalence of phylogroup B1 in domestic species (45); however, this was only true when comparing all wild hosts against domestic hosts and became insignificant when comparing only those which shared the same diet type (herbivores). Furthermore, PCA revealed that host status had the largest effect on within-sample phylogroup composition, suggesting a strong role for human influence on the domesticated animal gut with respect to E. coli diversity, potentially including direct treatment with antibiotics or exposure to human fecal biota.

Future investigations of E. coli diversity in wild hosts would benefit from the ability to identify individuals (e.g., by host-specific microsatellites) and track their movement before and after sampling; this would greatly improve our ability to investigate the influence of such host characteristics as body mass, home range, age, sex, etc., on within-host E. coli community composition and provide information regarding the different environments and thus the levels of human impact a host encounters. Although it did not impact the questions we addressed here, the opportunistic nature of our sampling methods prevented us from knowing whether each scat sample was from a different individual host. For example, based on puma characteristics, their range size, and current camera trapping data, it is known that no more than 5 individuals were on the preserve at a time, which implies that a single individual was likely sampled more than once over the duration of the study. Trap-and-release methods would address the difficulty of opportunistic sampling, especially for small mammals, and would also allow assessment of host health at the time of sample collection, as much remains unknown about the ability of E. coli strains which cause disease in humans to cause similar ailments across a diverse range of wild animals. Laboratory methods which enable investigation of the abundance of total E. coli present in each scat sample would inform a species’ potential to vector disease, as those with higher pathogen loads have a higher chance to infect other animals or humans. Assessments of seasonal variation in phylogroup distribution with long-term sampling protocols could evaluate the stability or transience of phylogroups within an individual wild host and would also inform spillover risk. The PERMANOVA analysis results showed significant differences in within-sample phylogroup community compositions between wet and dry seasons in California. Potential explanations for this observation could include seasonal changes in the host’s diet, indirect impacts to the gut microbiome of hibernation or decreased activity in winter months, or hydrodynamic effects such as concentrated water sources during dry summer months. However, more comprehensive genetic and functional studies are needed to disentangle the factors at play. It has also been suggested that fecal-based studies may underestimate strain diversity, which is partially attributed to the differential presence and abundance of E. coli along the intestinal track (46). Future studies would benefit from culture-independent methods to assess E. coli diversity, as it is still unknown whether certain E. coli phylogroups are more or less amenable to growth on solid medium, or whether strains that are significantly less abundant are capable of colony growth in the presence of other fecal bacteria. Despite these limitations, the PCR method used here to assign E. coli isolates to phylogroups is considerably less expensive than whole-genome sequencing (WGS), especially at the scale and sampling depth here, and allowed the rapid identification of genetically diverse E. coli strains that could then be selected for downstream WGS analyses to advance our understanding of evolution and host association in E. coli.

In contrast to prevailing methods investigating commensal E. coli diversity in wild hosts, which rely on a single isolate to ascertain within-host diversity, here, we sampled an average of 20 colonies per sample, thereby demonstrating that over half of them contained more than 1 phylogroup. Additionally, we sampled 7 different host species to phylogroup-level saturation. We found substantial phylogroup diversity both within individuals and across host species and showed that sampling comprehensiveness is a factor of phylogroup richness at the host-species level. These results suggest that observations surrounding the influence of certain host characteristics on phylogroup distribution are impacted by sampling intensity. This informs future work on E. coli in wild animals regarding the sampling depth necessary to uncover the true phylogroup diversity both within an individual and across a host species, and thus the genuine correlations between host characteristics and E. coli phylogroup distribution. There appears to be a wide range of host environments in which an E. coli phylogroup can live, implying that more investigations on the factors contributing to E. coli phylogroup community composition inside a wild host are needed. Continuing to expand our database on the genetic diversity of E. coli in wild animals through comprehensive sampling methods, complemented by WGS, will enable the development of better pipelines to assess host specificity by identifying regions of the genome that are more adept at strain assignment, thereby informing transmission routes, which is especially relevant to source-tracking contamination outbreaks. Our study implies that as thoroughly as we have studied E. coli as a model organism, we will be unable to predict pathogen emergence in food supplies or indeed in most human-wildlife interactions without a better understanding of the evolution and life history across all hosts and environments of this ubiquitous and highly genetically diverse bacterium.

## MATERIALS AND METHODS

### Study location.

Jasper Ridge Biological Preserve (JRBP) is located on Stanford University lands in the eastern foothills of the Santa Cruz Mountains in California, on the urban fringe of Silicon Valley. This partially fenced preserve encompassing 1,200 acres (5 km^2^) is composed of a striking diversity of habitat types, including serpentine grasslands, chaparral, oak woodland, and freshwater wetlands. It contains creeks, Searsville Lake, and a total of 34 marked and maintained trails and roads. JRBP has a long history of human influence, starting as early as 5,000 years ago with Native American occupancy and later experiencing extensive grazing and logging through the 1700 to 1800s. Searsville Dam was erected in the 1890s and the lake it created was used recreationally for the next 50 years, while the surrounding area hosted substantial levels of hiking and horseback riding. Following its designation as a biological preserve in 1973, the area was closed to the public, and today it supports a small staff at the field station and many important biological research projects. However, a trail on the northern-most border of the preserve is still used frequently for horseback riding and is proximal to horse stables. The eastern border of the preserve is directly adjacent to a cattle ranch, and the southern border abuts a residential area. Additionally, domesticated goats have been used for fire fuel reduction in designated areas of the preserve. The wildlife on the preserve has little to no contact with humans while on the preserve yet are free to come and go from the protected space.

### Sample collection.

**FIG 6:**
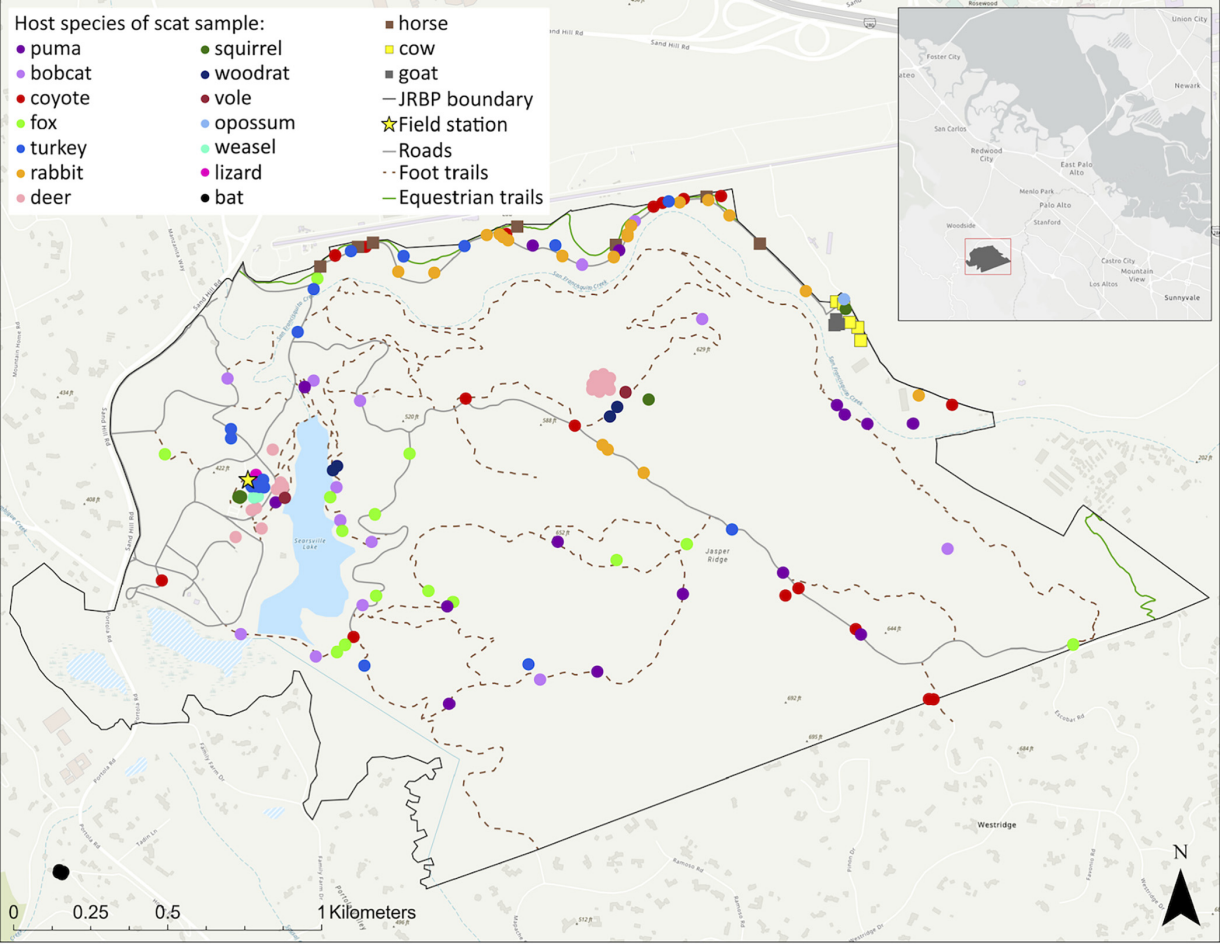
Study site: Jasper Ridge Biological Preserve. Collection points of all scat samples colored by their host species (ArcGIS Pro 3.0, ESRI, Redlands, CA). For map layer citations, see supplemental text.

Scat samples were opportunistically collected along trails throughout JRBP starting at the end of the dry season in October 2017 and continuing through April 2018 following a previously defined protocol (47). Prior to the start of the survey, all scat was removed along 34 paths (trails, 17 km; roads, 7 km) to ensure that the scat collected was no more than 2 days old. Sample collections continued after this initial procedure sporadically until the end of 2021, following the same protocol. Collection of fresh samples was occasionally guided by an extensive camera trap operation throughout the preserve (48). Upon collection, a photo of the scat was taken, its GPS location was recorded, and host species identification was made visually, if possible (Fig. 6). Other metadata recorded included ground cover type, level of sun exposure, and estimated age of scat by a ranking system (49) where 1 = fresh and of high quality (still moist, intact, and above leaf litter), 2 = intermediate quality and freshness (somewhat moist and intact), and 3 = low quality and older age (dry, easily broken, or beneath litter). Scat samples that were deemed too old or dry were destroyed *in situ* to ensure that no sample spent too long in the field prior to collection. Individual scat samples were collected in plastic bags with gloved hands to prevent cross-contamination. Samples were stored at −20°C until further analysis.

In total, 161 scat samples were used in this study from the following taxa: puma (Puma concolor; *n* = 17), bobcat (Lynx rufus; *n* = 16); coyote (Canis latrans; *n* = 20); gray fox (Urocyon cinereoargenteus; *n* = 14); black-tailed deer (Odocoileus hemionus; *n* = 18); turkey (Meleagris gallopavo; *n* = 19); rabbits (Lagomorpha; *n* = 21), primarily black-tailed jackrabbits (Lepus californicus) and Californian brush rabbits (Sylvilagus bachmani); California vole (Microtus californicus; *n* = 2); ground squirrels (Otospermophilus beecheyi; *n* = 6); long-tailed weasel (Mustela frenata; *n* = 2); dusky-footed woodrat (Neotoma fuscipes; *n* = 6); opossum (Didelphis marsupialis; *n* = 1); western fence lizard (Sceloporus occidentalis; *n* = 1); and bats (Chiroptera; *n* = 4) from a mixed-species bat roost 0.5 km from the preserve composed primarily of Mexican free-tailed bats (Tadarida brasiliensis) and big brown bats (Eptesicus fuscus). We also sampled 3 domestic species with occasional presence on the preserve, including horses (Equus caballus; *n* = 8), which use the equestrian trail on the northern part of the preserve; domestic cows (Bos taurus; *n* = 5), which broke down the fencing on the northeastern side of the preserve in November 2021 and so were only briefly present but live nearby; and a monitored goat herd (*Capra hircus*; *n* = 1 community sample) which was used for grazing as fire control in certain areas of the preserve in 2021.

### Host species identity confirmation.

If the host of a collected scat sample was in question after visual inspection, the host species was confirmed by swabbing the epithelial cells from the outer layer of the fecal sample with a synthetic cotton-tipped swab dipped in ATL buffer with light pressure to remove host cells and avoid fecal material. DNA extractions from these swabs were done using the Qiagen DNeasy blood and tissue kit (Valencia, CA). Minor modifications to the protocol included adding 25 μL proteinase K and incubating the sample at 56°C for 1 h and then at room temperature overnight before continuing with the extraction protocol, which involved separating solids from the lysis buffer, binding the DNA to silica in a spin column, and eluting in 50 μL of the provided elution buffer. Final eluted DNA concentrations were quantified by fluorometry and stored at −20°C until PCR amplification with the following MiMammal primers (50): MiMammal_F, 5′-GGGTTGGTAAATTTCGTGCCAGC-3′; and MiMammal_R, 5′-CATAGTGGGGTATCTAATCCCAGTTTG-3′. The PCR mix comprised a 20 μL volume: 10 μL of Promega GoTaq Colorless Master Mix (400 μM dATP, 400 μM 268 dGTP, 400 μM dCTP, 400 μM dTTP, and 3 mM MgCl_2_; Madison, WI), 1 μL of each primer, 4 μL of DNA extract, and 4 μL water. Cycling conditions used initial denaturing at 95°C for 10 min, followed by 35 cycles of denaturing at 95°C for 30 s, annealing at 60°C for 30 s, and extension at 72°C for 10 s. Following PCR, 7 μL of the reaction mix was sent for Sanger sequencing and species identification was made by performing NCBI Nucleotide BLAST on the resulting sequences.

### Escherichia coli isolation.

For large-quantity scat samples, 1 g of fecal material was taken from the interior of each scat sample to minimize environmental contamination and was homogenized in 9 mL water. For the smaller scat samples (deer, rabbit, ground squirrel, vole, woodrat, weasel, western fence lizard, and bat), approximately 0.5 g (the equivalent of approximately 3 deer or rabbit pellets; 4 to 5 woodrat, vole, or squirrel pellets; 8 bat pellets; or 16 lizard pellets) was homogenized in 4.5 mL water. Pellets were brushed or scrapped prior to inoculation to reduce environmental contamination. From these homogenizations, 4 serial dilutions of 100 μL each were plated on MacConkey agar (Thermo Fisher Scientific Inc., Waltham, MA), a selective and differential medium used to identify tentative E. coli isolates, and incubated overnight at 37°C. Red colonies were tentatively identified as E. coli and yield ranged from one to thousands of colonies per plate. The rabbit samples yielded no bacterial growth after the first plating, so 1 pellet each was homogenized in 1 mL PBS (phosphate-buffered saline) and incubated overnight at 37°C before plating again. This resulted in 3 positive samples. An average of 20 red colonies were selected per plate. All colonies were re-streaked for isolation and then inoculated in PCR tubes for downstream PCR analyses. The age classification of a scat sample did not affect E. coli yield: scat age class 1 (*n* = 36) yielded red colonies 58% of the time, class 2 (*n* = 46) 54% of the time, and class 3 (*n* = 79) 58% of the time. In total, 92 samples (57%) yielded red colonies on MacConkey agar (Table S1). Following molecular confirmation by quadruplex PCR (1), 84 scat samples (52%) were confirmed positive for E. coli, resulting in a total of 1,756 *E. coli* colonies. Of these scat samples, 71 were from wild animals: 156 E. coli isolates from 11 pumas (*P. concolor*), 240 isolates from 9 bobcats (*L. rufus*), 257 isolates from 13 coyotes (*C. latrans*), 223 isolates from 9 gray foxes (*U. cinereoargenteus*), 315 isolates from 12 black-tailed deer (*O. hemionus*), 146 isolates from 6 turkeys (M. gallopavo), 20 isolates from 1 rabbit (Lagomorpha), 10 isolates from 1 California vole (*M. californicus*), 46 isolates from 3 ground squirrel middens (*O. beecheyi*), 12 isolates from 1 long-tailed weasel (*M. frenata*), 10 isolates from 1 dusky-footed woodrat (*N. fuscipes*), 35 isolates from 1 opossum (*D. marsupialis*), 20 isolates from 1 western fence lizard (*S. occidentalis*), 11 isolates from 2 samples of the bat community composed of Mexican free-tailed bat (*T. brasiliensis*) and big brown bat (E. fuscus). A total of 13 samples were from domestic animals: 113 isolates from 7 horses (*E. caballus*), 91 isolates from 5 cows (B. taurus), and 51 isolates from a goat community (*C. hircus*).

We did not quantify the concentration of E. coli in each sample due to several factors, including the need to make many dilutions for some samples to prevent lawn growth while others needed overnight culturing in nutrient medium to grow. Some samples had high concentrations of white colonies, which could potentially have outcompeted or covered up the red colonies, and red colonies were not necessarily E. coli (for example, *Enterococcus* spp., Klebsiella spp., Raoultella planticola, etc.). Also, because *Shigella* spp. are technically E. coli but remain white on MacConkey agar, these were not included in this study. As a result, counting red colonies on a plate could overestimate or underestimate E. coli prevalence.

### Phylogroup assignment.

All isolates were classified into 1 of 8 phylogroups (A, B1, B2, C, D, E, F, G) or the cryptic clades by amplification of a small number of differentially present genes with a commonly used quadruplex PCR method (1, 2). The PCR mix comprised a 16 μL volume: 8 μL of Promega GoTaq Colorless Master Mix (400 μM dATP, 400 μM 268 dGTP, 400 μM dCTP, 400 μM dTTP and 3 mM MgCl_2_), 0.5 μL of each of the 4 primers sets (5 mM), and a single colony inoculated in 4 μL double-distilled water. The modifications to the protocol were few but included colony inoculation and reduction of the PCR mix quantity from 20 to 16 μL to preserve reagents. Cycling conditions used initial denaturing at 95°C for 2 min, followed by 35 cycles of denaturing at 95°C for 30 s, annealing at 55°C for 30 s, extension at 72°C for 20 s, and a final extension at 72°C for 5 min. DNA extracted from laboratory strain E. coli MG1655 was used as a positive control. Phylogroups C and E (ECOR strains) were obtained from a collaborator and used as positive controls in C-specific and E-specific PCR amplification. All reactions also included a negative control containing the reaction mix and no DNA template. DNA visualization was performed on 2% agarose gel. The age of the scat was determined not to be a factor in the number of phylogroups detected in a single scat sample (Fig. S3a).

### Statistical analysis.

All data analyses were performed using R Studio version 4.1.3 (Boston, MA) with *tidyverse* version 1.3.1 (51). The complete data table is included in the supplemental material (Table S2). Stacked bar charts were made with *ggplot2* version 3.3.6 (52) and reshape2 (53). Rarefaction curves were created with the ‘specaccum’ function in *vegan* version 2.6-2 (54) with the method set to “random” and 1,000 permutations. Linear modeling was conducted in R to assess the relationships between E. coli prevalence and the log of the average host species body mass in grams (55) and also between phylogroup saturation level and the log of the average individual home range size (km^2^) of each host species (56). We used Wilcoxon’s rank-sum test and the Bonferroni method to correct for multiple comparisons in R to test for differences between the relative abundances of the dominant phylogroups and host status and diet type.

To visualize the within-sample E. coli phylogroup composition, square root-transformed raw count data were visualized with PCA using PRIMER v6 software (57). Principal component axes 1 and 2 were plotted in *ggplot2* version 3.3.6 (52). PERMANOVA was performed using PRIMER v6 with Euclidean distance. The PERMANOVA model included 6 factors: host species (nested within diet), host diet (nested within status), and host status (wild or domestic) as a random effects; and collection year, season (wet or dry) at time of collection, and scat age as fixed effects. We ran a Type-III partial sum of squares PERMANOVA for 999 permutations of residuals. The variance explained was calculated by summing the estimated components of variation for the statistically significant terms and the residuals and dividing each by this total (58).

We used the Nestedness based on Overlap and Decreasing Fill (NODF) metric developed by Almeida-Neto et al. (59) to test for a nested subset pattern in E. coli abundance across 17 host species and used the null model c0 (60) to compare the observed data to 1,000 simulated random matrices, implemented using the ‘nestednodf’ and ‘ecosimu’ functions in *vegan* (54). NODF calculates a nested score for both rows and columns as well as for the whole matrix and is less prone to Type-1 errors than similar metrics (59). This allowed us to evaluate whether the whole matrix score was more affected by host species or E. coli phylogroup differences. To account for differences in sampling effort across host species, we randomly sampled an individual from each host species, ran the nestedness analysis with null model simulation to determine whether the subset phylogroup communities were more or less nested than random, then iterated 1,000 times.

### Data availability.

All data are available in the main text or the supplemental materials.
