# Supplementary material for: Under-Appreciated Phylogroup Diversity of Escherichia coli within and between Animals at the Urban-Wildland Interface
Source: Appl Environ Microbiol. 2023 May 16;89(6):e00142-23. doi: 10.1128/aem.00142-23 (PMC10305377; doi:10.1128/aem.00142-23)
Supplement: Supplemental file 1 — Supplemental material. Download aem.00142-23-s0001.pdf, PDF file, 1.4 MB [file aem.00142-23-s0001.pdf]

Supplemental Materials for

Under-appreciated phylogroup diversity of *Escherichia coli* within and between animals at the urban-wildland interface

Katherine M. Lagerstrom* and Elizabeth A. Hadly

This PDF file includes:

Figures S1 to S8

Tables S1

Supplemental Text

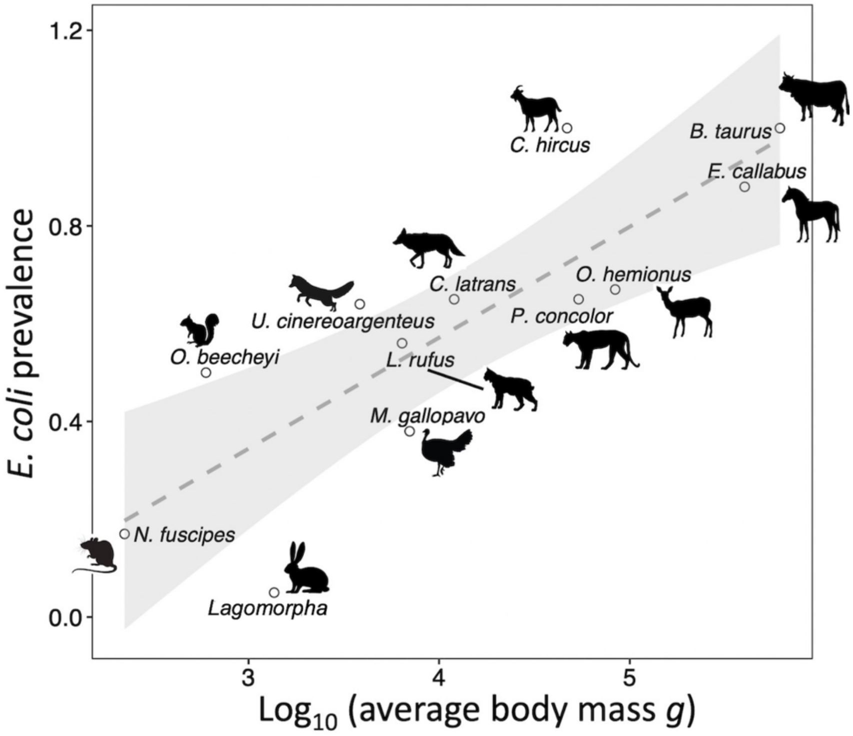

Fig. S1. Prevalence of *E. coli* among individuals within a host species increases with the average body mass of the host species. The y-axis is the proportion of all scat samples tested for each host species that yielded *E. coli*. Linear model formula: Host species' *E. coli* prevalence ~ $\log_{10}$(average host body mass *g*). Residual standard error = 0.1745, multiple R^2 = 0.6819, $P < 0.001$. Host species with n < 3 were excluded from this analysis.

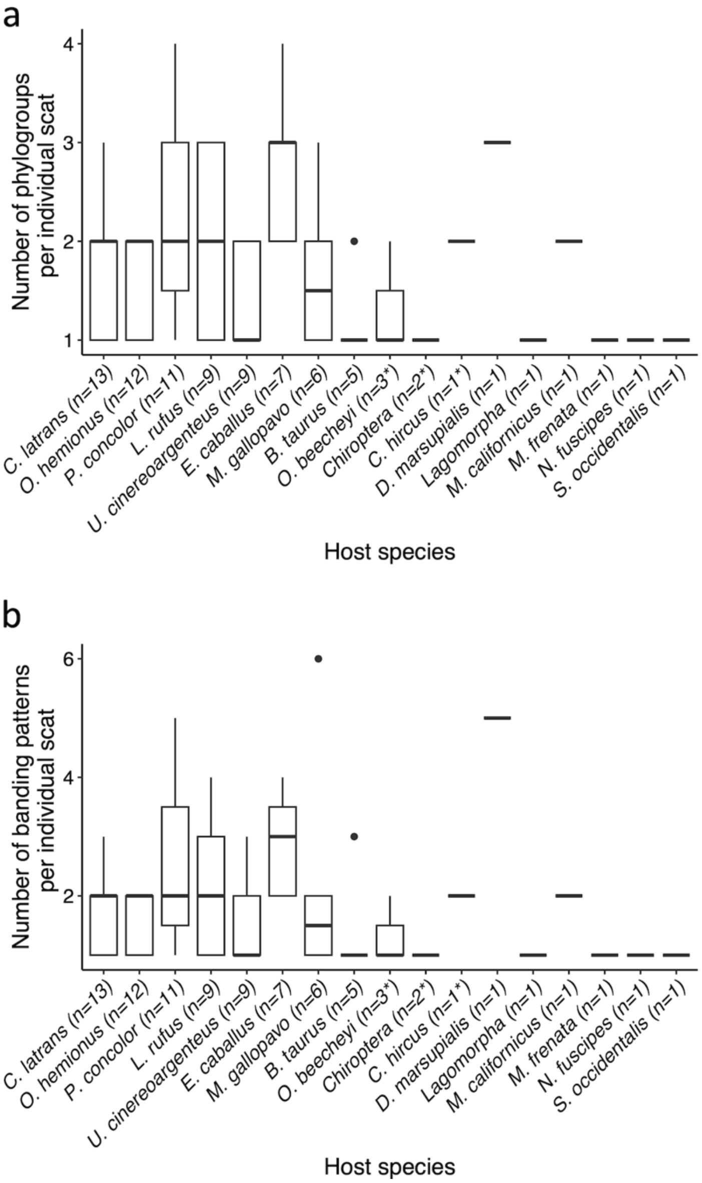

Fig. S2. Host species' within-sample *E. coli* phylogroup richness. Boxplots of (a) the number of phylogroups (of 9 including the cryptic clades) and (b) the number of unique banding patterns (of 14) obtained from each scat sample grouped by host species and listed in decreasing order of the number of individuals sampled per species. The dark horizontal line marks the median. *Denotes a multi-individual sample.

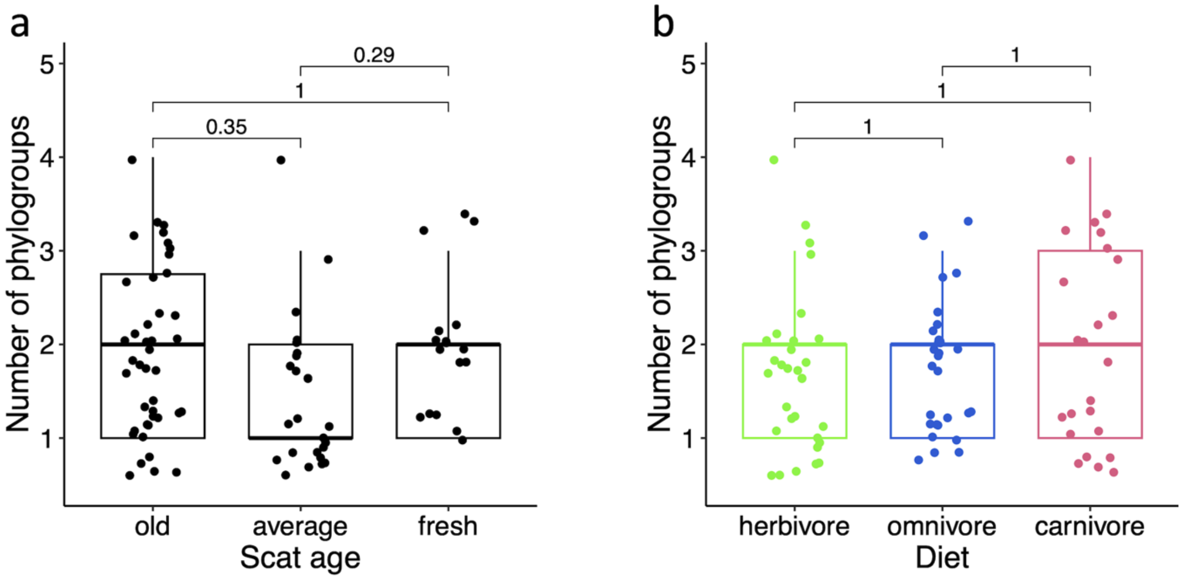

Fig. S3. Neither scat age nor host diet type affected within-sample *E. coli* phylogroup richness. (a) No significant differences were observed between the age assigned to a scat sample at the time of collection and the number of phylogroups obtained. (b) No significant differences were observed between the diet type of the host and the number of phylogroups isolated per scat sample. Scattered points represent individual scat samples. Mean comparisons were calculated with Wilcoxon's rank sum test corrected for multiple comparisons by the Bonferroni method.

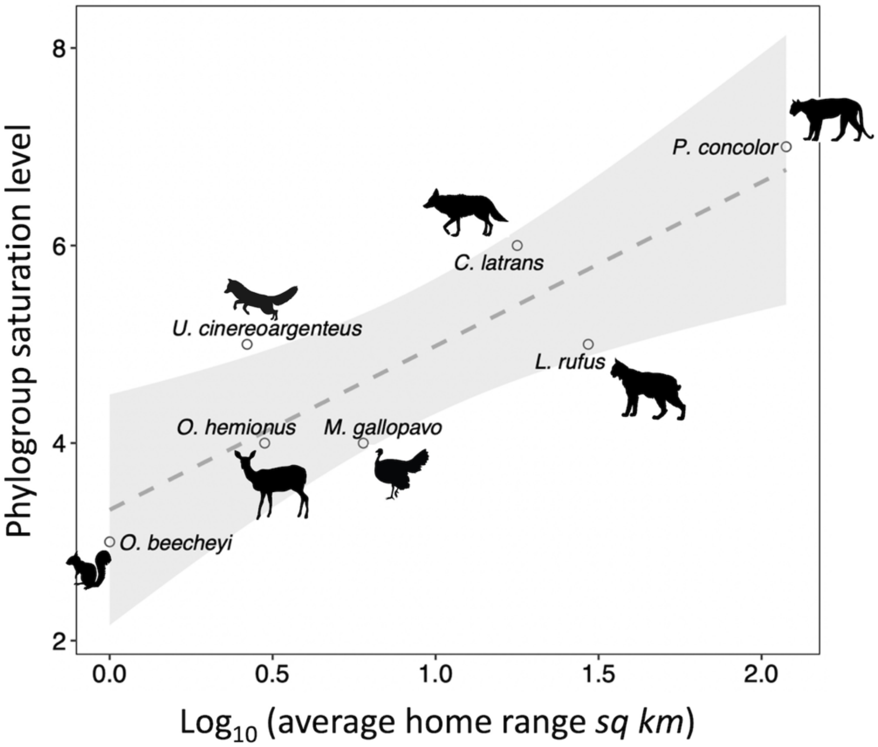

Fig. S4. The phylogroup saturation level of a host species increases with the average home range size of the host species. Data shown include the seven wild animal host species whose rarefaction curves reached saturation. Though sampled to saturation, the home range of a domestic horse is governed by humans, so horses were excluded. Linear model formula: phylogroup saturation level~ $\log_{10}$(1 + individual home range km^2). Residual standard error = 0.699, multiple R^2 =0.775, $P < 0.01$.

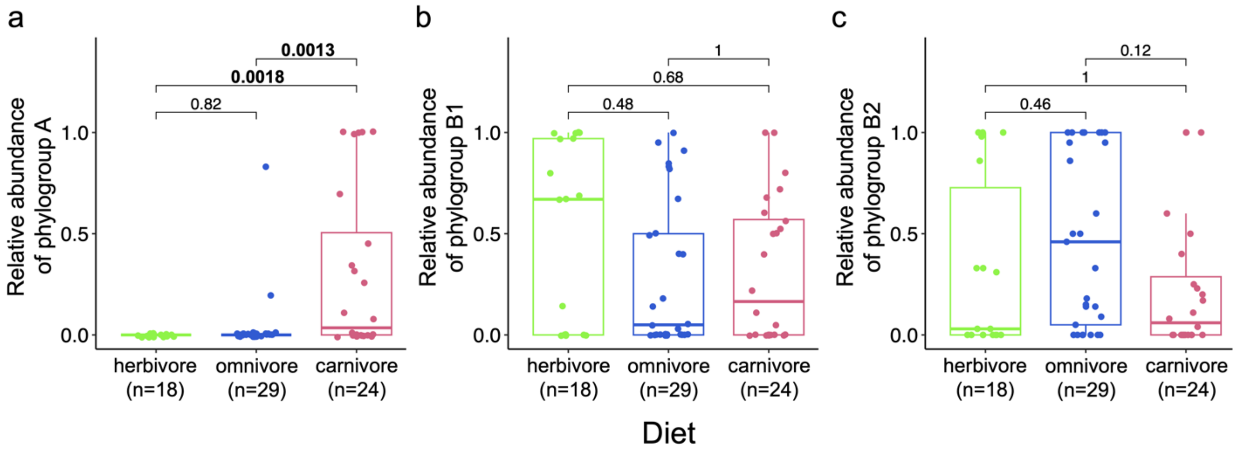

Fig. S5. Relative abundance of the dominant phylogroups between diet types of wild hosts. Scattered points represent individual scat samples, and the y-axis is the relative phylogroup abundance in each sample measured as a proportion of the total number of isolates taken from each sample. (a) Phylogroup A was absent in wild herbivores and was significantly more abundant in wild carnivores than wild omnivores or herbivores (Kruskal-Wallis; $P < 0.001$). There were no statistically significant differences in the abundances of (b) phylogroup B1 or (c) phylogroup B2 across diet types of wild hosts. Mean comparisons were calculated with Wilcoxon's rank sum test corrected for multiple comparisons by the Bonferroni method.

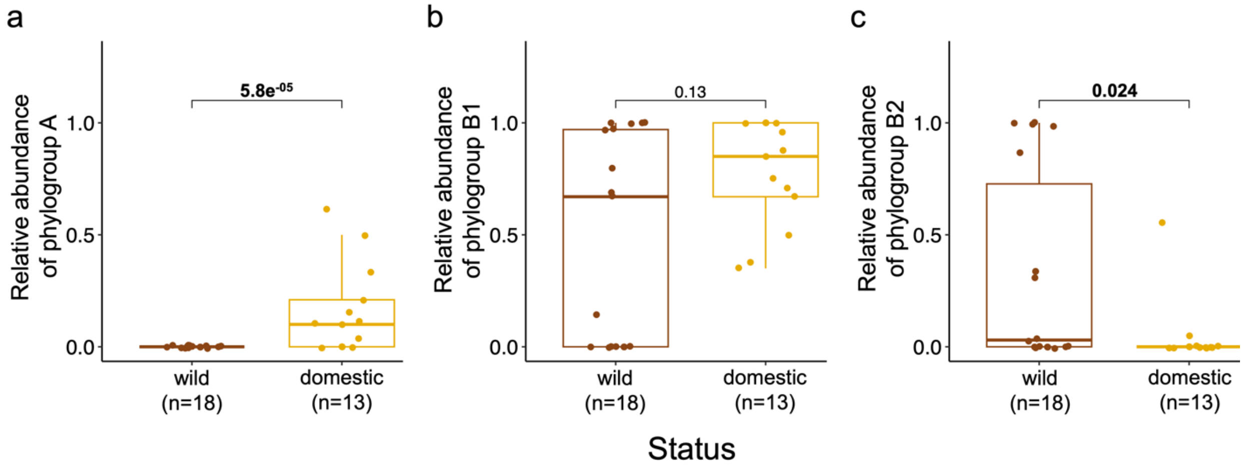

Fig. S6. Relative abundance of the dominant phylogroups between herbivorous wild and domestic hosts. Scattered points represent individual scat samples, and the y-axis is the relative phylogroup abundance in each sample measured as a proportion of the total number of isolates taken from each sample. (a) Phylogroup A was only present in domestic herbivores and not wild herbivores; (b) phylogroup B1 was slightly more abundant in domestic than wild herbivores, but not significantly so; and (c) phylogroup B2 was significantly more abundant in wild compared to domestic herbivores (Wilcoxon; $P < 0.05$). Mean comparisons were calculated with Wilcoxon's rank sum test corrected for multiple comparisons by the Bonferroni method.

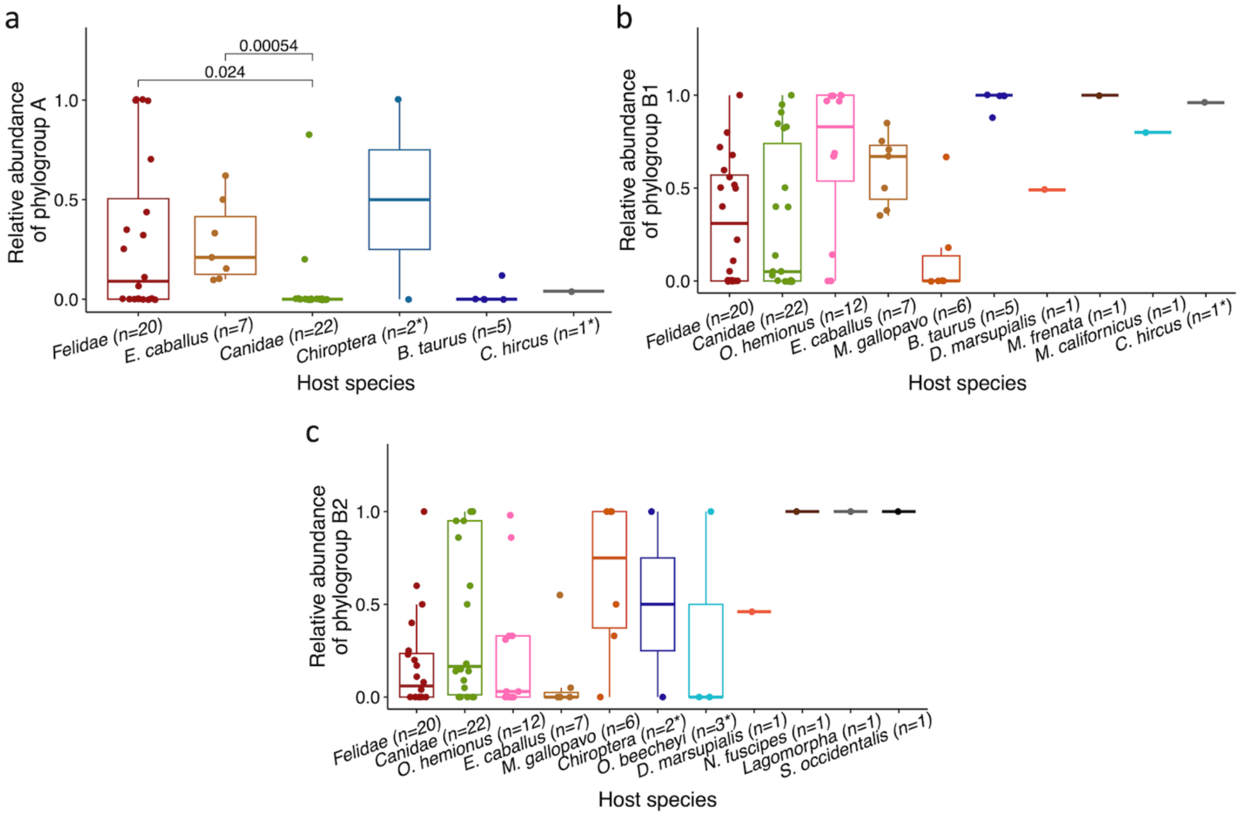

Fig. S7. Relative abundance of the dominant phylogroups across host species. Scattered points represent individual scat samples and relative abundance is measured as the proportion of isolates belonging to a phylogroup over the total number of isolates taken from that sample. (a) Phylogroup A was significantly more abundant in *Felidae* and horses than *Canidae* (Kruskal-Wallis; *P* < 0.01). Mean comparisons were calculated with Wilcoxon's rank sum test corrected for multiple comparisons by the Bonferroni method. (b) Relative abundance of phylogroup B1 (Kruskal-Wallis; *P* < 0.01); and (c) phylogroup B2 across host species (Kruskal-Wallis; *P* > 0.05). *Lynx rufus* and *Puma concolor* (*Felidae*) and *Canis latrans* and *Urocyon cinereoargenteus* (*Canidae*) were grouped to account for phylogenetic relatedness. Host species with no individual carrying that phylogroup are not shown on the associated graph.

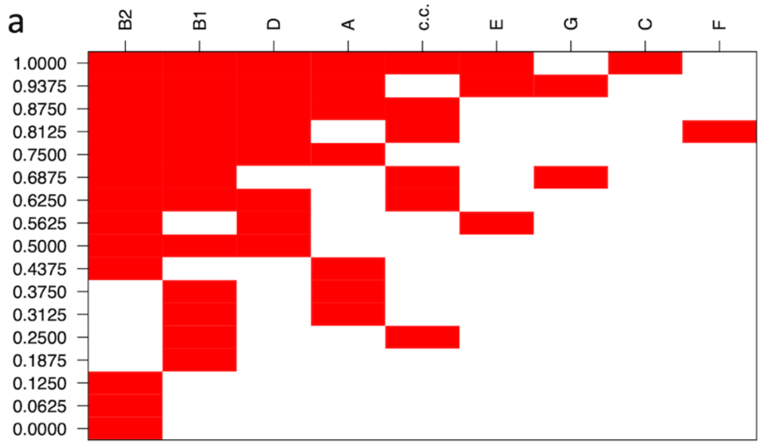

	statistic	SES	mean	50%	95%	Pr(sim.)
N columns	69.957	3.5897	50.514	50.562	59.515	0.000999***
N rows	67.794	3.0107	50.438	50.613	59.585	0.001998**
NODF	68.247	3.3739	50.454	50.578	58.983	0.001998**
Matrix fill	0.3464052					

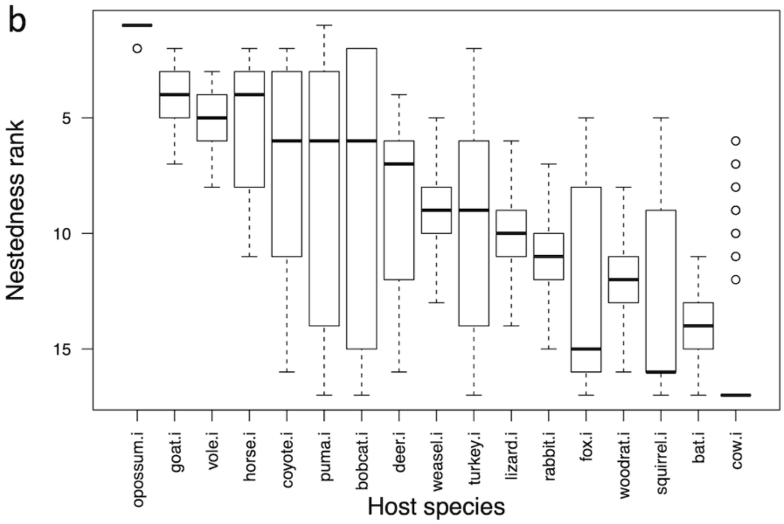

Fig. S8. Nestedness analysis results. (a) Packed nested matrix of occurrence of each phylogroup within each host species. (b) Average nestedness rank for each host species after correcting for the number of individuals sampled per host species by random selection of an individual from each host species for the nestedness analysis with null model simulation to determine if the subset phylogroup communities were more nested or less nested than random, iterated 1,000 times.

Table S1. Prevalence of *E. coli* across host species and diet types. Scat samples yielding red colonies on MacConkey agar were considered tentatively positive for *E. coli*. False positives were those samples that yielded red colonies that subsequently were not confirmed as *E. coli* by PCR. These are accounted for in the difference between the values in columns 'Tentative' and 'Confirmed.'

**Host species**<br>* multi-individual sample	**Total no. of scats**	**Tentative positive n (%)**	**Confirmed positive n (%)**	**Diet**	
				**Herbivores**	
				Total n	Confirmed positive n (%)
Lagomorpha	21	3 (14)	1 (5)	67	31 (46)
Odocoileus hemionus	18	13 (72)	12 (67)		
Microtus californicus	2	1 (50)	1 (50)		
*Otospermophilus beecheyi**	6	3 (50)	3 (50)		
Neotoma fuscipes	6	3 (50)	1 (17)		
Bos taurus	5	5 (100)	5 (100)		
*Capra hircus**	1	1 (100)	1 (100)		
Equus caballus	8	7 (88)	7 (88)		
				**Omnivores**	
				Total n	Confirmed positive n (%)
Urocyon cinereoargenteus	14	9 (64)	9 (64)	54	29 (54)
Canis latrans	20	13 (65)	13 (65)		
Didelphis marsupialis	1	1 (100)	1 (100)		
Meleagris gallopavo	19	9 (47)	6 (32)		
				**Carnivores**	
				Total n	Confirmed positive n (%)
Lynx rufus	16	9 (56)	9 (56)	40	24 (60)
Puma concolor	17	11 (65)	11 (65)		
Mustela frenata	2	1 (50)	1 (50)		
*Chiroptera**	4	2 (50)	2 (50)		
Sceloporus occidentalis	1	1 (100)	1 (100)		
**Total**	**161**	**92 (57)**	**84 (52)**		

Supplemental Text

Fig. 5 references:

Jasper Ridge Biological Preserve (Agency). 2017. Jasper Ridge Biological Preserve Boundary (Polygon). [Shapefile]. Retrieved from https://earthworks.stanford.edu/catalog/stanford-mf819md0352

Stanford University. Herbert, T. 2022. Hiking and equestrian trails at Jasper Ridge Biological Preserve. Stanford University, CA, USA. Retrieved from https://services.arcgis.com/7CRlmWNEbeCqEJ6a/arcgis/rest/services/JRBP_Transportation_CorCorrid/FeatureServer
